# Supplementary figures and images for: Teropavimab and zinlirvimab sensitivity in people living with multidrug-resistant HIV-1: data from the PRESTIGIO Registry
Source: Microbiol Spectr. 2025 Sep 11;13(10):e02777-24. doi: 10.1128/spectrum.02777-24 (PMC12502735; doi:10.1128/spectrum.02777-24)

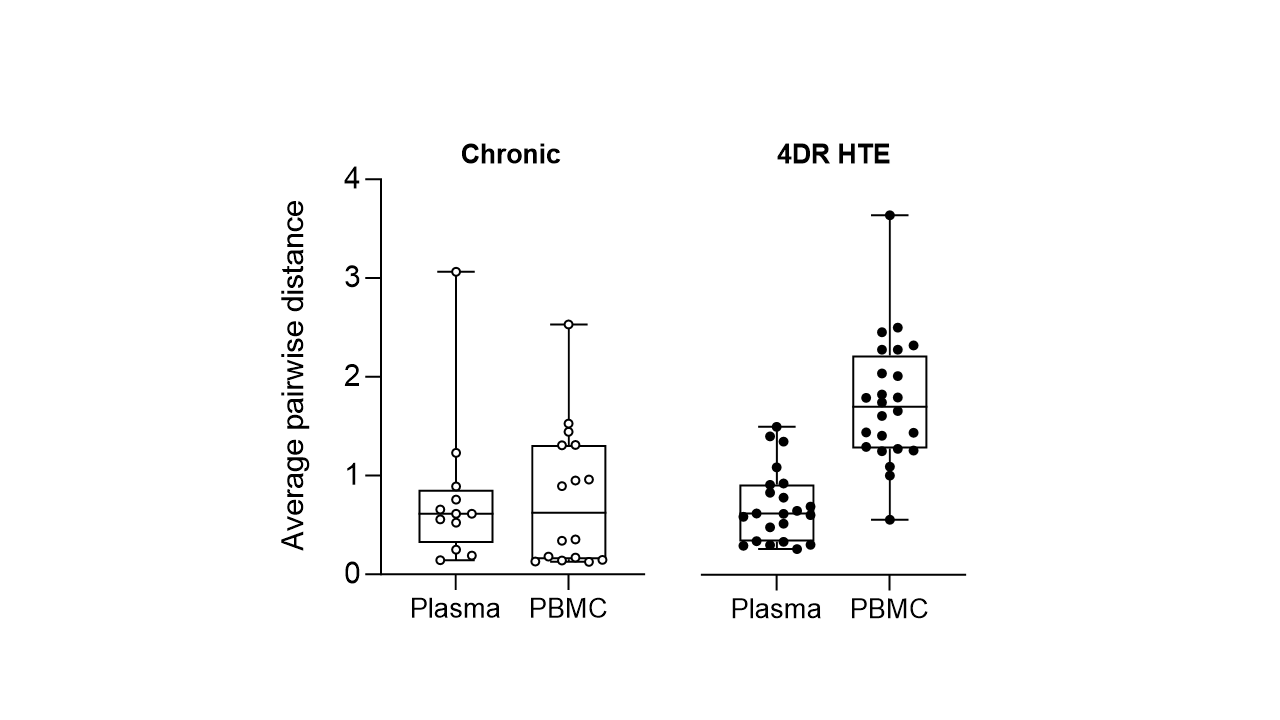

Supplement: Fig. S1 — HIV Env sequence diversity in PWH. [file spectrum.02777-24-s0001.tif]
