# Supplementary material for: Teropavimab and zinlirvimab sensitivity in people living with multidrug-resistant HIV-1: data from the PRESTIGIO Registry
Source: Microbiol Spectr. 2025 Sep 11;13(10):e02777-24. doi: 10.1128/spectrum.02777-24 (PMC12502735; doi:10.1128/spectrum.02777-24)
Supplement: Supplemental material — Fig. S1 legend. [file spectrum.02777-24-s0002.docx]

**Supplementary Figure.** HIV Env Sequence Diversity in PWH. Diversity was evaluated by average pairwise distance for plasma virus and PBMC provirus from all participants. Data shown as box and whisker plots including all data points; Diversity of HIV env sequenced from plasma and PBMC virus from individuals in clinical trials who initiated ART during chronic infection (>6 months) included for comparisons [18,19]
